# The Stream Exchange Protocol: A Secure and Lightweight Tool for Decentralized Connection Establishment

**DOI:** 10.3390/s21154969

**Published:** 2021-07-21

**Authors:** Stefan Tatschner, Ferdinand Jarisch, Alexander Giehl, Sven Plaga, Thomas Newe

**Affiliations:** 1 Fraunhofer Institute AISEC, 85748 Garching bei München, Germany; ferdinand.jarisch@aisec.fraunhofer.de (F.J.); alexander.giehl@aisec.fraunhofer.de (A.G.); sven.plaga@aisec.fraunhofer.de (S.P.); 2 Department of Electronic and Computer Engineering, University of Limerick, V94 T9PX Limerick, Ireland; thomas.newe@ul.ie; 3 Department of Informatics, Technical University of Munich, 85748 Garching bei München, Germany

**Keywords:** peer-to-peer, federated, trust model, secure connection establishment, connectivity

## Abstract

With the growing availability and prevalence of internet-capable devices, the complexity of networks and associated connection management increases. Depending on the use case, different approaches in handling connectivity have emerged over the years, tackling diverse challenges in each distinct area. Exposing centralized web-services facilitates reachability; distributing information in a peer-to-peer fashion offers availability; and segregating virtual private sub-networks promotes confidentiality. A common challenge herein lies in connection establishment, particularly in discovering, and securely connecting to peers. However, unifying different aspects, including the usability, scalability, and security of this process in a single framework, remains a challenge. In this paper, we present the Stream Exchange Protocol (SEP) collection, which provides a set of building blocks for secure, lightweight, and decentralized connection establishment. These building blocks use unique identities that enable both the identification and authentication of single communication partners. By utilizing federated directories as decentralized databases, peers are able to reliably share authentic data, such as current network locations and available endpoints. Overall, this collection of building blocks is universally applicable, easy to use, and protected by state-of-the-art security mechanisms by design. We demonstrate the capabilities and versatility of the SEP collection by providing three tools that utilize our building blocks: a decentralized file sharing application, a point-to-point network tunnel using the SEP trust model, and an application that utilizes our decentralized discovery mechanism for authentic and asynchronous data distribution.

## 1. Introduction

Currently, secure communication schemes for the exchange of information are more important than ever. The growing complexity of the internet in respect of subnetting, firewall techniques, Network Address Translations (NATs), Software Defined Networking (SDN), and Virtual Private Networks (VPNs), complicates the implementation of these technologies. At the same time, this complexity also hinders the general reachability of single devices [1].

A good example are Internet Service Providers (ISPs), who operate private networks for mobile internet connectivity that limit the general reachability of connected devices. To overcome such barriers for remote access or maintenance work, different techniques are employed to establish network connections. These, however, often provide workarounds but do not address the real problem of device reachability. In the following, we use the term smart devices for Internet of Things (IoT), Smart Home, Smart Lighting, or similar network capable applications.

Addressing the requirements of non-experienced end-users, most smart devices are designed for minimal user interaction. Therefore, they provide their functionality out-of-the-box with no additional configuration efforts. Consequently, smart devices need to be provided with robust and secure connection establishment techniques to create stable and ultimately secure network connections in untrusted environments. Recent applications (cf. Section 2) prefer clients communicating directly to other clients in order to exchange information, referred to as Peer-to-Peer (P2P) communication [2,3,4,5,6].

In this topology, peers can be mediated through a central server if they are not able to establish direct connection. The mentioned central server essentially adopts the position of a Man-in-the-Middle, which—in the absence of end-to-end security—requires each client to fully trust this server. Moreover, decentralized communication techniques can be used to distribute network traffic, reduce the load on central components, and ensure availability [7,8].

Previous studies highlighted that the current cloud-based approach used by smart devices has scalability issues due to the lack of proper abstractions [9]. Vendors still provide central servers either operated by themselves or through a third party providing additional functionality. Video surveillance systems use cloud platforms enabling access to the caputured material, smart home platforms use proprietary infrastructure offering remote configuration capabilities, and media streaming applications use central services connecting peers at any location together [10,11,12]. Using these features, the user has to entrust third parties with handling their data, which might result in unwanted data breaches.

Further work shows that proper security concepts do not appear to have a high priority in development [11]. The authors revealed that smart devices often use proprietary or misconfigured protocol suites leading to vulnerabilities, which allow remote attackers to eavesdrop or conduct Man-in-the-Middle attacks. As observed on some smart devices, they might not implement any security mechanisms at all, leaving them completely vulnerable to attacks [13,14]. Due to the lack of a generic and secure framework, vendors repeatedly implement individual concepts for connection establishment and repeatedly make the same mistakes [15,16,17,18,19]. As a consequence, five shortcomings were identified as part of this work: *complexity*, *modularity*, *maintainability*, *scope*, and *insecurity*.

To overcome these shortcomings, five design goals were specified: *simplicity*, *generality*, *practicability*, *scalability*, and *security*. From these design goals, the Stream Exchange Protocol (SEP) collection of secure, generic, and reusable building blocks for decentralized connection establishment was created. SEP generalizes existing work in the form of building blocks whose modular design encourages developers to also reuse only parts of them. In order to encourage discussion and eventually create a community with a growing ecosystem, we provide a reference implementation as a library.

On top of the mentioned library three small utilities were developed that thoroughly utilize the previously presented concepts: a decentralized file sharing application (*SEPHole*), a point-to-point network tunnel using the SEP trust model (*SEPTun*), and an application that utilizes our decentralized discovery mechanism for authentic and asynchronous data distribution (*blobber*).

## 2. Related Work

Various related technologies share design challenges in terms of re-usability and universality. As a first step, we analyzed free (as in free speech) technologies that create network tunnels of which some were designed for particular use cases, for instance file synchronization. In order to derive our own design goals, we focused on the disadvantages of the selected technologies and grouped them in five different categories of shortcomings:

- **Complexity.** The software is overly complex and difficult too use. It is easy to make mistakes that lead to, e.g., poor confidentiality.
- **Modularity.** The software lacks modularity that limits re-usability in further projects.
- **Maintainability.** The software is difficult to maintain, since there are conceptual flaws or many distinct infrastructure components are needed.
- **Scope.** The software is limited in scope and difficult to deploy in large environments.
- **Insecurity.** The security of the software is not part of its design.

A relevant technology is the Syncthing [3] project, which “replaces proprietary sync and cloud services with something open, trustworthy and decentralized”. The project offers decentralized file synchronization with automatic peer discovery and NAT traversal. The central infrastructure required for NAT traversal is provided by the project team free for everybody to use.

However, if the central infrastructure suffers from a Denial-of-Service attack, every peer will be unreachable except from within the local network. Additionally, high load at those central parts of the infrastructure impairs the performance of each Syncthing device. It is possible to create and manage one’s own infrastructure that is independent from the servers of the Syncthing project. The design does not implement the concept of federation, which means a group of computing or network providers agree upon standards of operation in a collective fashion.

Consequently, data replication between individually operated infrastructures is not supported and they remain isolated from each other. This inadequately manifests in the usage of Syncthing device-IDs, which carry no domain information and are global in scope. Suffering from these fixed structures, the community often experienced service degradation issues in the past. While the Syncthing developers reacted by adding new servers to their central infrastructure, the fundamental issue of poor scalability remains unresolved [20]. SEP improves peer discovery by using a standardized fingerprint data format and adding an authority part to the fingerprints. The authority is used as an infrastructure component to distribute authenticated data enabling federation (cf. Section 5.1).

In order to protect the transport layer, Syncthing uses an up-to-date Transport Layer Security (TLS) protocol revision (at the time of writing version 1.3). Trust is established by exchanging a Syncthing specific fingerprint between the participants. On mobile devices, this is implemented using QR-Codes, which is a user friendly and accepted used concept. The Syncthing protocol collection consists of several sub-protocols for discovery, NAT traversal, and data transfer. As each of these sub-protocols has been designed in accordance to the Syncthing specification, re-usability is limited. SEP solves this problem with a modular design where each protocol component can be used on its own. This modular design is eventually used by the developed tools to prove its versatility. Overall, the Syncthing project suffers from the shortcomings *modularity*, *maintainability*, and *scope*.

The Magic Wormhole [5] is an easy-to-use file transfer tool that automatically creates a secure channel between two peers through NATs and firewalls. It enables two parties to establish a short-lived P2P connection trusted by exchanging a temporary, human readable password string. A proprietary protocol is used, which implements the key exchange mechanism on the SPAKE2 algorithm [21] negotiating a shared secret for the libsodium-based [22] transport phase.

The program appears well-engineered, secure, and fulfills its purpose out-of-the-box. However, the architecture suffers from scalability issues: A central “Rendezvous-Server” is required in order to perform the key exchange and discover IP-addresses in order to establish a P2P connection, “The URL of [this] public server is baked into the library [...] until volume or abuse makes it infeasible to support” [5]. The project does not offer re-usability, as its design limits use cases to file transfers. Using a modular design with the option of combining different infrastructure components, SEP is not affected by these problems. The Magic Wormhole project suffers from the shortcomings *modularity*, *maintainability*, and *scope*.

The libp2p library [6] is a general purpose library that was factored out of the Interplanetary Filesystem project [23] in order to create a reusable collection of modules. SEP and libp2p share the same basic idea; however, their approaches differ greatly in detail. The libp2p project modularized every possible component in the relevant network stack, such as: transports, discovery, routing, and even security-relevant parts. Since all these modules can be combined together arbitrarily, this comprehensive modularization approach seems overly complex.

Considering the project documentation, it becomes apparent that the transport layer is not designed around security, since it is possible to disable protection completely. In Chapter 3.3 of the requirements document the project team states: “We recognize that encryption is not viable for some in-datacenter high performance use cases”. This design choice is risky, since decentralized network protocols may reroute traffic and leak confidential data outside their designated domain. Therefore, the libp2p is not sufficient for confidential data relying on the security of the communication protocol. By using mandatory secure default settings and a simpler design, SEP is not affected by the same problems. The libp2p project suffers from the shortcomings *complexity* and *insecurity*.

The *BitTorrent* protocol [24] was one of the first implementations of a distributed P2P file sharing protocol. Exclusively designed for file sharing, confidentiality and authenticity were not design goals. To address these shortages, some proposals for security extensions have been made. One of these drafts proposes a signature method to verify the authenticity of a torrent issuer. While the topic of confidentiality is targeted by another proposal, the presented scheme was found to be not very effective.

Therefore, confidentiality is still left to underlying secure transport protocols, such as VPNs [7,24]. Since the BitTorrent protocol is designed around file sharing, the protocol is also not suitable for generic purposes. SEP reuses some ideas from the infrastructure components, i.e., the trackers as components at well-known network locations ease the distribution of metadata. Consequently, SEP distributes required metadata using directories that serve authentic data distributed by nodes. The BitTorrent project suffers from the shortcomings of *modularity* and *insecurity*.

The *WireHub* [2] project is a recent project that emerged around the Wireguard VPN. Wireguard claims to be “an extremely simple yet fast and modern VPN that utilizes state-of-the-art cryptography” [25] and is part of the Linux kernel. WireHub automates peer configuration, which is already kept at a minimum by only relying on exchanging encoded public key strings, which is similar to the approach of Syncthing. Furthermore, WireHub provides automatic peer discovery and NAT traversal capabilities based on Distributed Hash Tables (DHTs).

Since WireHub is based on a DHT and built upon the Wireguard VPN protocol its use case is too specific for general re-use. SEP reuses ideas around its simple configuration and automatic peer discovery but takes a more modularized approach to be generally applicable. The WireHub project suffers from the shortcoming *modularity*.

All identified shortcomings are summarized in Table 1.

In contrast to the related work, SEP targets a more general and yet simpler approach. By following appropriate design goals, SEP was designed to avoid the aforementioned shortcomings from the beginning. Therefore, its design is evaluated so that the presented related technology can be re-implemented transparently for the application using SEP.

## 3. Design Goals

In the following section, the design goals and the philosophy behind the SEP suite are presented. These are the result of investigation of related work including the identification of demands and weak spots within the specifications of current technologies. Our design goals strive to overcome the identified shortcomings of the presented related work.

1. **Simplicity.** The SEP collection aims to be easy to use and lightweight, while keeping the overhead at a minimum. For a developer, it must be obvious how to use the protocol rather than being confronted with an abundance of choices. Thus, strong security mechanisms are built in and always enabled. For a user of SEP-enabled applications, it must be easy to understand how trust is managed across multiple peers. Additionally, it must be virtually impossible to make implementation or usage mistakes that lead to catastrophic security breaches. The purpose of this design goal is to avoid the shortcoming and its associated aftermath of *complexity*.
2. **Generality.** The protocol collection is designed to be as general and reusable as possible. Therefore, it implements a secure and decentralized transport layer with no application-specific semantics. It is designed to be usable from the beginning of new design processes yet also adaptable for legacy deployments. The purpose of this design goal is to avoid the shortcoming *modularity*.
3. **Practicability.** The SEP suite is designed to be fully decentralized with the possibility to centralize certain components. This increases the availability, interoperability, and robustness of the overall design. With this federated design, peers can be grouped into logical zones that are able to discover peers belonging to different zones. The required infrastructure is kept at a minimum and available technologies are reused as much as possible. The purpose of this design goal is to avoid the shortcoming of *maintainability*.
4. **Scalability.** The concept of trust is straightforward for just two communication partners, as well as manageable for a large number of peers. Much like the Domain Name System (DNS), inter-domain functionality must be possible where multiple infrastructures with data replication are combinable by design. Furthermore, it is possible to update or replace parts of the protocol collection in order to create an evolving and improving ecosystem. The purpose of this design goal is to avoid the shortcoming of limited *scope*.
5. **Security.** The SEP suite is intended to be a robust protocol suite protecting its users by design with secure default settings. For this purpose, we shielded the suite against an attacker who has full access to network packets that are transferred between two peers. In that assumed attacker model, an  adversary is able to eavesdrop on the communication channel, manipulate sent packets, or inject additional packets. However, access to peers themselves and particularly to any secrets, such as private keys, is not within the attacker’s capabilities. The purpose of this design goal is to avoid the shortcoming of *insecurity*.

## 4. Concept Overview

The Stream Exchange Protocol was designed as a network protocol collection for *establishing* secure P2P network connections. Its main purpose is being an easy to use middleware for both developers and users of SEP applications. SEP provides transport functionality by managing the underlying network communication as well as secure transport aspects. Trust management, all aspects of transport security, and P2P connection establishment are handled by SEP and kept away from the application layer. In other words, SEP is considered to be the baseline for higher level protocols, since SEP itself implements no application logic.

**Figure 1:**
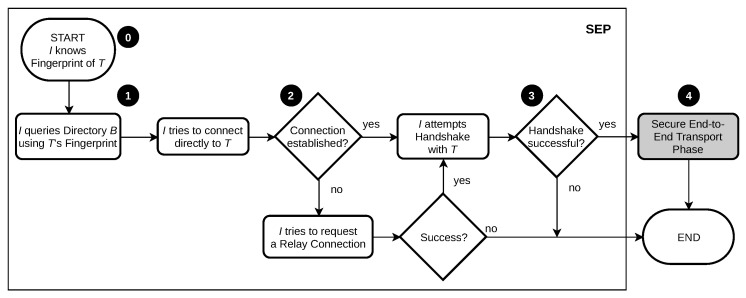
Schematic flow chart of the process of connection establishment of a secure end-to-end channel within the SEP suite.

To provide a general summary of the main purpose of the SEP suite, Figure 1 provides an overview. The numbers of the following explanation refer to the numbers in the figure. In this example, a node (initiator *I*), initiates a connection to another node (target *T*), if necessary via a relay *R*. The connection establishment based on the mutual authentication of each connected node is highlighted. Fingerprints, which carry enough information to uniquely identify and authenticate a peer, are assumed to be exchanged prior to the connection attempt. These fingerprints are derived from a cryptographic public key and are explained in Section 5.1. To explain certain aspects of the communication flow, the key steps are numbered and explained below:

0. In order to initiate a connection establishment, node *I* needs to know the fingerprint of node *T*. Actively creating the connection, the initiator trusts this fingerprint. This does not necessarily have to be the case the other way around.
1. Utilizing the target’s fingerprint, the initiator can locate the responsible directory and discover the record set that was previously announced by node *T*. The signature of the record set is validated, such that the end-to-end authenticity of the announced data is ensured. With the information contained in the record set, node *I* now attempts to establish a direct network connection to node *T*.
2. If the initiator is not able to establish a direct network connection, a relay is optionally utilized to reach the target. From the perspective of node *I*, trust towards the relay is inherited from the target due to the signature of the record set. From the relay’s perspective, trust has to be earned by the approval of the relay request by node *T*. If this succeeds, the relay starts with passively forwarding network traffic, allowing the initiator to attempt a handshake with the target.
3. In case the target is not aware of the initiator’s fingerprint, the handshake fails and further connection attempts are denied. Node *T* accepts the incoming connection only if trust was established beforehand by the mutual exchange of fingerprints.
4. A secure end-to-end connection is established according to the chosen transport connection setup. This means that the transport phase can start and actual application data is being transferred within the established secure channel. The scope of SEP ends at this stage and appropriate technologies perform this task, e.g., TLS.

For the design goal Security, we address the protection goals of mutual authenticity between two peers, and the integrity and confidentiality of transferred data, as further described below. SEP does not specifically account for the anonymity of peers, as fingerprints are designed to be permanently attributed to a single peer, and data announced by this peer can be queried by anyone knowing its fingerprint. Moreover, the availability of peers (i.e., their fingerprints) is not addressed apart from generic decentralization within the overall design. Robustness or speed, for instance, can be improved with multiple discovery techniques or by parallelizing connection attempts.

- **Trust Management.** Trust between two peers trying to establish a connection is mandatory and needs to be built beforehand by exchanging fingerprints, which loosely translates to the exchange of public keys of a public/private key pair. Information necessary for connection establishment received in step 1 may be stored on directories but is then required to be signed with the corresponding peer’s private key. Utilizing these signatures, trust can also be dynamically passed on to other fingerprints contained within signed data.
  It is assumed that fingerprints are exchanged outside of SEP via a secure channel that guarantees integrity.
- **Self-certified Connection Establishment.** In order to tackle attacks during connection establishment, we utilize self-certified fingerprints to identify peers [26]. These fingerprints carry enough information to uniquely identify and authenticate a peer, and moreover provide information on how to locate it. As fingerprints need to be exchanged prior to connection establishment, the initiating peer is able to abort corrupt connection attempts, either in the case of invalid signatures in step 1 or in case the target can not authenticate itself during the handshake in step 3. As opposed to this, the target peer rejects all incoming but untrusted connection attempts, which results in the process described above always failing at step 3.
- **Secure Default Settings.** To prevent attacks on established connections, only well-proven standard protocols, which guarantee confidentiality, integrity, and authenticity are utilized with pre-defined settings, such as the specific ciphersuite. This does not unnecessarily increase the attack surface by creating own implementations and prevents users from creating faulty or exploitable configuration settings.

## 5. Architecture

**Figure 2:**
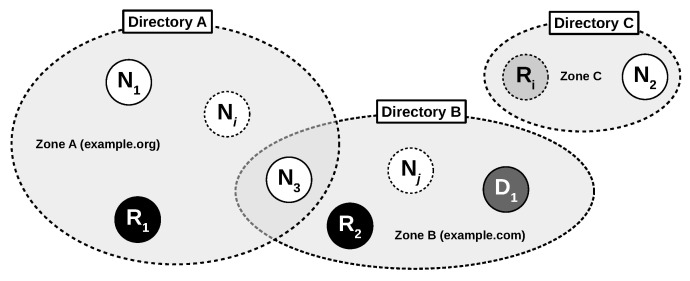
Overview of SEP’s decentralized ecosystem exemplified by three different zones and multiple nodes in different roles.

Figure 2 shows an architectural overview of the Stream Exchange Protocol’s ecosystem. Except the directory, each party involved during the life cycle of an SEP connection is a node, and any node can adopt the special role of a relay or a delegator, which can be described as follows:

- **Node** **(N).** Nodes are entities that are capable of running the Stream Exchange Protocol. They own an asymmetric key pair representing their unique identity. Initially, there is no declaration of trust among distinct nodes, and trust needs to be established by exchanging fingerprints. A node trying to establish a connection is called the initiator (I), while the corresponding node is called the target (T).
- **Relay** **(R).** This node forwards network traffic between two other nodes and, thus, can facilitate connectivity beyond different (sub-)networks or gateways. After assisting the establishment of a secure end-to-end connection, a relay only forwards end-to-end encrypted traffic.
- **Delegator** **(D).** This node can be entrusted by other nodes to perform dynamic trust management. Configuration overhead in terms of exchanging fingerprints for trust establishment or updating trust relationships is reduced using delegator nodes.

While most of the SEP components are optional, the directory is mandatory as it stores data for nodes that associate themselves with that particular directory. Thus, an administrator can build a zone *A*, such as example.org, by running a zone directory *A* and instructing connected nodes to associate with that directory. This allows nodes to announce their current network location, available protocols, or relays $R_{1},R_{2},\ldots ,R_{i}$ where they expose themselves. A zone is defined as the group of nodes that announce to the same directory. Apart from the cost of maintaining multiple fingerprints, there is no limitation for nodes announcing their information to multiple directories at the same time.

The protocol collection consists of multiple functional components, where each can exist on its own, or be replaced transparently. In addition to classic addressing schemes known from TCP or UDP, SEP contributes novel concepts for unique identities, addressing, and node discovery. For the compatibility with existing infrastructures, SEP utilizes existing technologies where applicable. In the following, we present the four building blocks identified for creating and managing end-to-end secured connections in the shown architecture:

- **Unique** **Identities.** Each node $N_{i}$ has a unique identity—called its fingerprint. This identity is the most basic building block and is used in various parts of the protocol as it represents the required information for both addressing *and* authentication of nodes. Encoded to a single fingerprint, this scheme, thus, allows locating and authenticating a node by only using its self-certified fingerprint. Consequently, fingerprints are the only information a node must store to be able to successfully verify a connecting peer.
- **Node** **Discovery.** The only component an organization must provide in order to form a zone of SEP nodes is a directory. Each node can join a zone by regularly updating the corresponding directory with a record set that contains the node’s current network location, available relays and other data. Other nodes can then query the directory for certain fingerprints and discover the data that was announced earlier. Conceptually, a directory supports two operations:
  1. **Announcement.** This operation is performed regularly by each node in order to dynamically push information in the form of a record set to the directory. Each record set is signed by the announcing node to guarantee the integrity and authenticity of the record set.
  2. **Discovery.** This operation is performed prior to connection establishment in order to resolve a node’s fingerprint into its recently announced record set. This allows other nodes to gain information about its current network location, available relays, and additional data.
- **Connection** **Establishment.** This building block is an essential part of SEP. In addition to direct peer-to-peer connection attempts, it can be extended to use relays automatically. This becomes relevant when two nodes are in distinct (sub-)networks separated from each other. Connection establishment can dynamically occur within or between multiple zones, as zones are not segregated from each other within SEP.
- **Transport.** This building block provides the state where an SEP connection is established and the delivery of application data starts. The transport building block exists in order to provide an interface for applications to ease development. Due to this design, multiple transport protocols, such as TLS, can be utilized.

### 5.1. Unique Identities

Identities are the most important building block in the Stream Exchange Protocol. Therefore, the remaining components are built on top of it. A node’s identity is called a *fingerprint*. Fingerprints are encoded using Named Information (ni) URIs [27] providing a set of standard methods to use hash function outputs in names. Such an ni URI contains an authority component and a hash value (val) bundled with an ID (alg) describing the applied hash algorithm.

The authority component defines which zone directory the node announces itself to and, consequently, allows other nodes to identify and locate the relevant directory. The supported hash algorithms are registered in the Named Information Hash Algorithm Registry [28]. It specifies a broad range of hash algorithms where some are presumed to be outdated and truncated and, in fact, too short to fulfill the property of uniqueness. As the fingerprint is supposed to serve as an *unique* identifier, we decided to limit the options to the sha3-256 hash algorithm.

**Figure 3:**
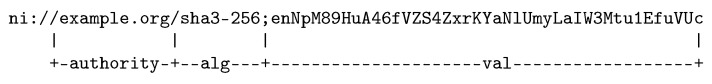
Example of an SEP fingerprint.

The actual hash value is generated by applying the hash function to the Distinguished Encoding Rules (DER) encoded [29] public key of the node’s key pair. To avoid problematic characters, the result is subsequently encoded in the base64 url-safe variant. Since information about the hash function is part of the fingerprint, the URI scheme stays forward compatible once new hash algorithms are added or old ones are considered deprecated due to security concerns. The resulting fingerprint of a node is shown in Figure 3.

The fingerprint is an abstract notation that allows discovering data that was previously announced by the corresponding node, such as the current network location. Once a node proves ownership of the corresponding private key, the fingerprint can be used to validate the authenticity of a node’s public key and, thus, the node itself. The ownership of the private key can be proven by, e.g., a valid signature of a record set or a successful TLS handshake. This allows considering the fingerprint to be self-certified.

The authority component of the fingerprint is not relevant for authentication, as a node can dynamically announce to multiple directories at the same time. As a consequence, two fingerprints are considered equal, as long as both the hash algorithm and hash value match. To enable the mutual trust between two nodes, fingerprints must be exchanged out-of-bounds prior to the first connection attempt. Fingerprints carry the relevant information for both authentication and addressing and are, thus, interlinked by design.

A SEP connection can only be established if both parties trust each other, and this trust needs to be built by exchanging fingerprints. With an increasing number of nodes, maintaining trust relationships between nodes can quickly become a burden for the administrator, as each node needs to store fingerprints of trusted peers individually. Thus, modifying fingerprints requires an administrator to update every single node that is supposed to interact with that modified fingerprint. In a fully meshed peer-to-peer network, this means that the administrator has the burden of reconfiguring the total number *N* of participating nodes.

For use cases where zones with a large number of nodes are maintained by a single administrator, e.g., a network of IoT sensors, the role of a delegator to alleviate the administrative burden is proposed. Delegators are nodes that can be entrusted by any other node to automate fingerprint distribution. Consequently, a node that delegates trust management to a delegator is required to trust only a single fingerprint, identifying the connected delegator.

The node, then, pulls additionally trusted fingerprints dynamically from the delegator and stores them in a non-persistent database. This automatically extends the list of trusted peers for the node. Thus, adding or revoking trusted fingerprints of a node can easily be done on the delegator, which reduces the administrative burden of reconfiguring nodes separately. As a consequence, however, nodes that delegate trust management fully rely on the trustworthiness and integrity of the delegator.

The list of trusted fingerprints on the delegator is stored separately for each individual node, such that modifying trusted fingerprints of one node has no implications for other nodes. On the delegator, however, an administrator can add multiple fingerprints to an abstract group and, thus, quickly implement group-based access policies. These group policies are then translated to individual lists of trusted fingerprints for each corresponding node.

### 5.2. Node Discovery

Node discovery requires a directory that can be queried for information stored there by other nodes in the first place. Thus, the directory must support two operations, *Announce* and *Discover*, where nodes either push or pull information. In order to create an SEP zone, an organization must provide a directory. Once announced, the node is associated to the directory. Conceptually, there is no limitation for nodes in the number of directories they can announce to. In the following sections, we describe the concept of the directory and present the design choices of our reference implementation.

The directory maintains the information that is published by the associated zone’s members. Existing technology such as Syncthing or The Magic Wormhole have a bootstrap problem. A participating node must connect to the relevant infrastructure first in order to be able to gain knowledge of further participating nodes. The related work solves this problem by providing a fixed list of entry points, which is used to initially join the network.

In order to avoid the mentioned bootstrap problem, SEP was designed to use existing infrastructure. The address of the responsible directory is encoded in a node’s fingerprint, for instance using DNS names or IP addresses. Due to the reversed data flow of the directory’s two operations *Announce* and *Discover*, they are differentiated in the SEP architecture. This guarantees that each operation can be implemented on its own, hence, enabling useful features, such as distinct permission rules.

**Figure 4:**
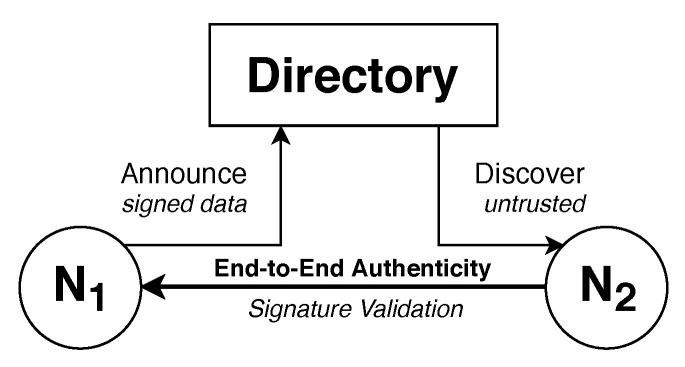
End-to-end authentication concept of the directory.

Figure 4 illustrates the architecture of the directory, which provides two endpoints that are connected to an internal key-value database. This database associates a node identified by its fingerprint with recently announced information called a record set. Such a record set carries information in multiple data types referred to as data records. End-to-end authenticity between two nodes interacting with a directory can be verified due to a signature of the entire record set added by the announcing node $N_{1}$ that can be verified by the discovering node $N_{2}$.

The Announce endpoint requires node $N_{1}$ to sign incoming record sets and to verify the signature before storing the record set in the database. This verification is based on the unique fingerprints each node possesses and the corresponding key pair that is used to establish a secure channel for the information transport. The Discover endpoint does not require connecting nodes, such as $N_{2}$, to authenticate themselves, as the stored record sets are considered public information. A connecting node, however, can only discover the record set of a certain fingerprint and can never generically retrieve all fingerprints known to the directory.

Table 2 shows a single record set containing one data record of each possible data type that the directory must be able to handle. Data types marked with an asterisk are mandatory and unique for a record set, while unmarked data types can occur zero, single, or multiple times. In the following, the available data types and their purpose are described.

#### 5.2.1. Address

The address data type corresponds to A or AAAA records known from DNS with the addition of the underlying transport protocol, such as TCP, and the corresponding port where the announcing node is listening for incoming connections. The URI format was used to encode this kind of information, since it is a widespread standard and parsers are available in most programming languages. Using the URI format, the notation of an address record is expressed as follows: tcp://198.51.100.42:1234 for IPv4 or tcp://[2001:0DB8::1]:1234 for IPv6. This data record specifies a node that is reachable on IPv4 address 198.51.100.42 or IPv6 address 2001:0DB8::1 and listening on TCP port 1234.

#### 5.2.2. Relay

In order to locate relays where a particular node exposes itself, the record set provides a relay data record that contains the fingerprint of the relay. Utilizing this fingerprint, a connecting node can establish an SEP connection to the relay just as it would with any other node and then continue to connect to the initial target. Since the announcing node signed the entire record set, the discovering node can also trust the retrieved relay fingerprint by following the chain of trust.

#### 5.2.3. Blob

To add generic versatility, the directory should accept a Blob data type that can contain any binary data that the announcing node wants to include. As this opens up the possibility of resource exhaustion, the directory must implement a size limit for the Blob record. Developers can use this Blob data record to easily define custom data types and develop new protocols around the concepts of SEP. If necessary, additional native data types can be added to directories in the future.

#### 5.2.4. Timestamp and TTL

In order to allow discovering nodes to evaluate the freshness of a record set and to avoid stale record sets, each set includes a timestamp of the time when the record set was initially created and a Time-to-Live (TTL) chosen by the announcing node. To ensure that only up-to-date records are submitted, the directory must verify the signature of the record set and reject invalid or timed-out record sets with an error. Similarly, the directory must continuously monitor its database and drop timed-out record sets. Generally, nodes are encouraged to maintain and update their corresponding record set in the directory by re-announcing themselves periodically. For a local network, the TTL should be kept at a minimum (i.e., a few seconds) in order to reduce the attack surface for replays. For global networks, it is possible to extend the TTL up to a few hours.

#### 5.2.5. PubKey and Signature

In addition to the aforementioned data types, the announcing node has to add the public key corresponding to its fingerprint and has to sign the entire record set. This signature is generated by the announcing node by sorting, combining, hashing, and signing all data records with its private key. With the signature covering the PubKey record as well, the announcing node proves ownership of the corresponding private key. As the public key is included in the signed record set, any node can validate the signature and, thus, the integrity of the record set. This means that the Discover endpoint itself is not required to be trusted, since the authenticity of served data can be end-to-end validated at the application layer. Furthermore, the discovering node can validate whether the initially queried fingerprint matches the corresponding hash of the public key included in the record set, which creates a self-certified discovery process.

### 5.3. Connection Establishment

This building block covers the process of connection establishment, which enables nodes to actually establish end-to-end protected P2P network connections. In general, the directory provides the IP addresses of a node such that establishing direct connections should be straightforward. However, complex network structures and restriction policies, e.g., implemented by firewalls with enabled NAT, aggravate connection establishment (cf. RFC2993 [30]).

As zones in SEP describe an abstract concept that lies on top of real network structures, not even nodes in the same zone are guaranteed to be able to directly communicate with each other. Thus, a mechanism is needed to facilitate NAT traversal and expose nodes to increase their reachability—namely relays.

A relay itself is an SEP node that is capable of all the aforementioned building blocks and additionally allows negotiating a relay connection between an initiating node (initiator) and a target node (target). Relays can be hosted and utilized regardless of the initiator’s or target’s zone to provide nodes with an option for NAT traversal [1]. As SEP focusses on end-to-end protected connections between two nodes, relays are not part of the secure session. Instead, they are simply forwarding network traffic once an end-to-end connection is established.

**Figure 5:**
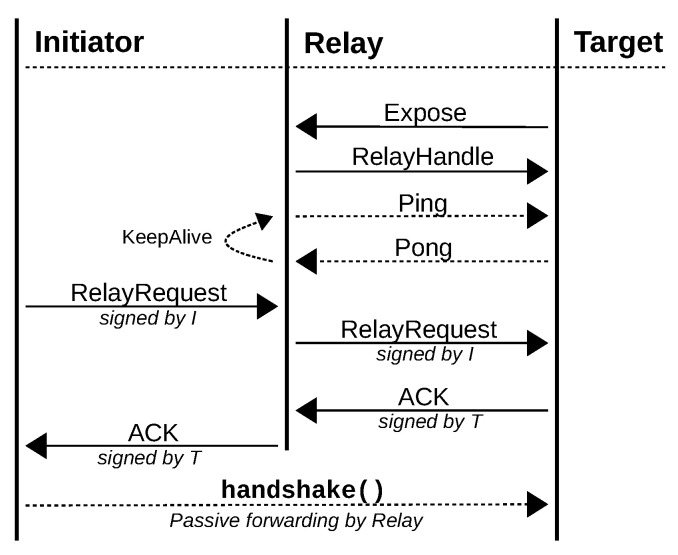
Protocol flow of a relay connection.

The protocol flow of a relay connection is visualized in Figure 5. At first, the target (T) establishes a SEP connection to the relay and, thus, actively exposes itself. The relay and the target maintain an active network connection by exchanging ping messages regularly, e.g., in order to maintain the state in firewalls. Now, being exposed to the relay, the target can announce the relay’s fingerprint to its directory, such that other nodes gain knowledge about the relay.

This allows an initiator (I) to automatically connect to the relay once direct connection establishment failed, and issue a relay request containing the fingerprint of the target. Once the forwarded request is acknowledged by the target, the relay drops active communication with both the initiator and target and starts acting as a passive forwarder of network packets. Finally, the initiator and target start a handshake and establish a logical and secure SEP connection via the relay.

Establishing an SEP connection requires both nodes, initiator and target, to know each others fingerprint beforehand for mutual end-to-end authentication. Not only does the initiator know the relay’s fingerprint from discovering the target but also trust is inherited from the target due to a valid signature of the record set. From the relay’s perspective, however, the initiator is unknown and, thus, untrusted. Consequently, the relay has to accept an untrusted connection at first but must put it into quarantine and must drop the connection if the target does not acknowledge the relay request within a short time delay. In other words, an initiator cannot negotiate a relay connection if it is not trusted by the target and the relay does not unnecessarily expose the target.

Relayed connections are not conceptually limited to a single hop and can be chained if needed, such that the connection to the relay of the target is established via a second relay. A high hop count, however, might cause unpredictable performance and latency issues as the classic IP-based internet is the basis for SEP connections.

### 5.4. Transport

The transport block is the last building block involved in the entire connection establishment process (cf. Figure 1). It is responsible for transferring user data. According to the design goal of *generality*, SEP is designed as a middleware and, therefore, allows being used with different network communication protocols, such as TCP or UDP. Usually, these basic transport protocols are provided by the network stack of most modern operating systems.

**Figure 6:**
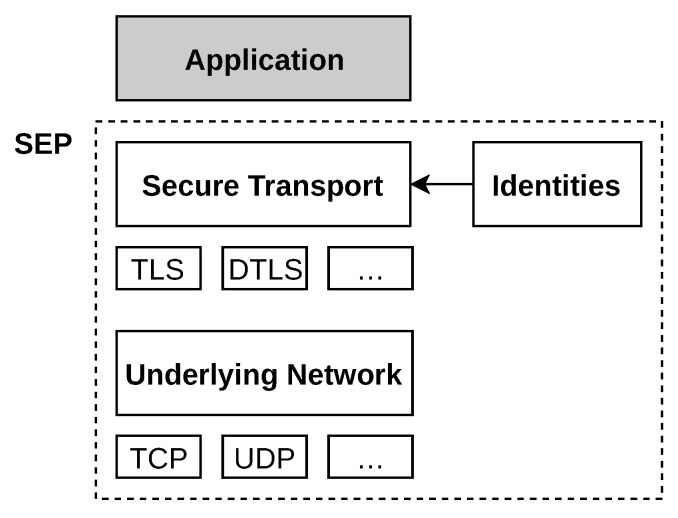
Exemplary overview of SEP in the network stack.

Each transport protocol, however, has different properties and provides different connection semantics, such as reliable or unreliable connections. Therefore, different standards for securing these basic communication primitives exists. For securing TCP connections, the well known TLS protocol suite and its DTLS counterpart for UDP are good examples. Due to its wide deployment, TLS has been analyzed in great detail over the years [31,32]. In the past several attempts of formal verification of security protocols have been conducted [33]. Being designed as a middleware, SEP provides a unified interface for a secure connection establishment process based on a combination of different transport protocols and security measures, as visualized in Figure 6.

Identities in the form of fingerprints provide all information required for the secure transport interface to establish mutually authenticated communication channels. Thus, the initiator can query the directory with the fingerprint of the target, which provides recently announced address records with active communication endpoints. Since address records encode transport protocol and address information using the URI standard, multiple associated endpoints can be distinguished, enabling interoperability and forward compatibility.

We propose a transport building block to be implemented with TLS on top of TCP and calculated fingerprints from the public key contained in self-signed certificates. Authentication on both sides is performed by hashing the public key from the peer’s TLS certificate and comparing the hash value with a list of known fingerprints. Since a TLS-based implementation obtains the required information from the TLS handshake, no further application specific message formats are needed. For VPN use cases, we propose a transport block based on UDP and DTLS.

## 6. Implementation of Node Discovery

The developed reference implementation of the directory system utilizes two standard technologies for the Announce and Discover endpoints: HTTPs and DNS. In the following, we describe this implementation and a few design choices, which are based on the format of ni URIs.

### 6.1. Announce via HTTPs

In our reference implementation, we use client certificate authentication in the TLS handshake between the node and the directory using the public key of the announcing node. The directory server subsequently computes the node’s fingerprint from the public key of the TLS client certificate and verifies the signature of the record set. Transfer of the record set is technically realized as a HTTP PUT request carrying a JavaScript Object Notation (JSON)-based payload. The entire record set is then inserted into a database using the connecting node’s fingerprint as the lookup key.

### 6.2. Discover via DNS

The distributed and multi-zone architecture of SEP can be naturally mapped to the semantics of the Domain Name System, which provides a hierarchical and globally decentralized naming system. Being the foundation of the internet for decades, DNS is available in almost all networks and provides multiple levels of caching, which improves the latency and distributes the server load globally. Due to its age, unfortunately, it is, by default, not protected by any security measures. However, by utilizing the described end-to-end authentication of the node discovery process (cf. Figure 4), this does not manifest itself as a problem. Moreover, the increasing deployment of other security measures, like Domain Name System Security Extensions (DNSSEC) [34], adds another layer of security.

**Figure 7:**
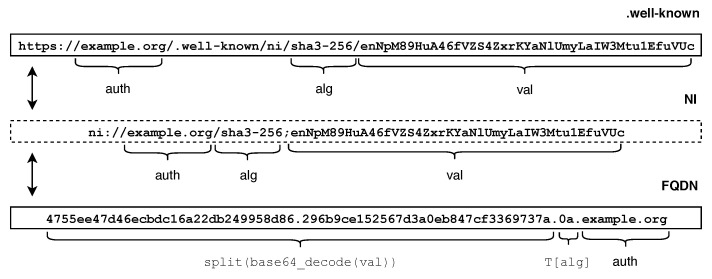
Fingerprint transformation from ni to FQDN and from ni to .well-known format.

In order to integrate DNS as an implementation of the Discover endpoint into SEP, we developed a logical extension to RFC6920 [27] converting ni URIs to an appropriate Fully Qualified Domain Name (FQDN) format that is compatible to the DNS name scheme. The relationship between a fingerprint in ni and FQDN notation is visualized in the bottom of Figure 7.

According to the ni standard, a fingerprint consists of four pieces of information: the scheme (*ni://*), the authority (auth), the applied hash algorithm (alg), and the value in base64 encoding (val). The scheme is not used in the FQDN format and can be dropped. While the auth part is kept unmodified, the alg and the val part need to be transformed to subdomain labels to be compatible to DNS names.

- T[alg] performs a table lookup and converts the algorithm field to a two digit hexadecimal number in ASCII encoding as specified in [28].
- split(base64_decode(val)) applies the base64 decoding operation to val and, due to size restrictions of subdomains, splits the resulting hexadecimal string in ASCII encoding by inserting an ASCII dot at every 32nd character. This operation is performed in the reverse direction, such that the first subdomain might be shorter than 32 characters.

The resulting FQDN can be queried via the standard DNS infrastructure enabling global data distribution and caching, which provides TXT records that carry key-value pairs in ASCII encoding according to Table 2.

### 6.3. Discover via HTTPs

Being in widespread use and well understood, HTTPs qualifies as a fallback protocol if, e.g., DNS is restricted by the local network. As RFC6920 [27] already defines a mapping scheme for converting the ni URI scheme to HTTP URLs, another implementation of the directory utilizing HTTPs is possible. To add another layer of security to the existing end-to-end authentication of the node discovery process (cf. Figure 4), we decided to change the protocol to https. This adds integrity, authenticity, and confidentiality to the discovery process. Thus, the relevant parts of an ni URI only need to be filled in a string template, which is defined in the standard as visualized in the top of Figure 7.

**Figure 8:**
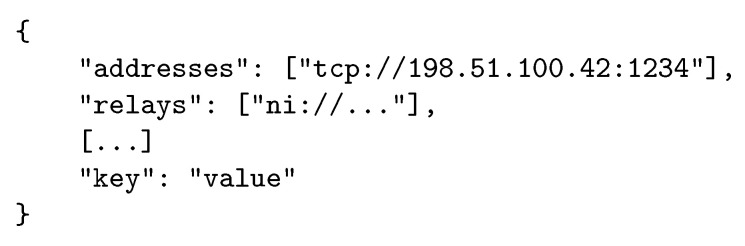
Scheme of a JSON serialized record set.

Nodes pull record sets from the directory over https using HTTP GET requests with a JSON-based encoding scheme. Since the directory is organized as a key-value store with strings as keys and values, the mapping to a JSON scheme is straightforward (cf. Figure 8).

## 7. First Use Cases

To show the general versatility of the Stream Exchange Protocol, three applications utilizing various building blocks of SEP were developed. The first use case is a file sharing application called *SEPHole* with a design and user experience similar to the Magic Wormhole [5]. The second utility, *SEPTun*, is a simple implementation of a VPN program that uses the concepts of SEP for the networking related code. The third application reuses the Blob record of the directory record set in order to implement an authenticated and decentralized data distribution service—named *blobber*.

*SEPHole* is an application for securely sending files between two peers. As a proof-of-concept, it utilizes the entire SEP software stack with all four building blocks. As presented earlier in the paper, two peers can establish a mutually authenticated TLS connection simply by exchanging their respective fingerprints, which reduces the required user interaction to a minimum. From a developer’s perspective, the code of the SEPHole is limited to application layer code (cf. Figure 6), which only requires a handful of calls to the SEP library. In a nutshell, all required steps, such as communicating with a directory server for announcement and discovery, signature validation of record sets, and optional NAT traversal utilizing relays, are handled by SEP. Due to its simple design, only three library calls are necessary to generate a key, announce the node to a directory, and establish an authenticated connection.

*SEPTun* is a small utility to create point-to-point network tunnels using the SEP trust model. Using SEP as a middleware for connection establishment, trust management, and transport protocol abstraction, this tool is versatile and yet simple. With one library option in the application-specific code, it is possible to change the underlying network stack from TLS over TCP to DTLS over UDP. Due to the library design, node discovery, connection establishment, and trust model aspects share the code and stay the same for both options. The implementation of SEPTun proves that the modular design of SEP is sound and works in practice.

The *blobber* utility exemplifies the idea of having multiple, reusable building blocks that are usable discretely. Consequently, the tool does not establish a peer-to-peer connection to other SEP nodes but only relies on the building blocks of *Unique Identities* and *Node Discovery*. Utilizing the Blob record of the record set, the blobber allows easy announcement and authenticated discovery of arbitrary data. Thus, decentralized data distribution services of authenticated data can be build on top of the SEP infrastructure. Similar to the SEPHole, only one library call is required to either distribute or retrieve data, once a TLS key pair is generated. Overall, the blobber is a relatively simple tool that can, for instance, be used to asynchronously distribute authentic sensor data or share PGP public keys.

## 8. Conclusions

With the Stream Exchange Protocol collection, we presented a new approach for establishing secure and decentralized network connections. The motivation for this work initially arose from the dissatisfaction with insecure IoT devices on the market. Needing to be accessible with minimal user interaction, those devices and corresponding applications kept re-implementing similar concepts that were often lacking security characteristics.

Moreover, we found that existing solutions specifically targeting peer-to-peer communication often incorporated built-in application characteristics that restricted their re-usability. To overcome these problems, we built the Stream Exchange Protocol suite on five design goals that were based on the identification of weak spots within current technologies and the demand for future concepts.

The first design goal, bringing Simplicity in a complex topic, is a complex task. We accomplished this goal by designing the whole toolset around the concept of identities and self-certified fingerprints. From a user’s perspective, the only required information to establish a secure connection is the mentioned fingerprint, as it is used to both discover and authenticate the target. A developer or administrator on the other hand can easily create a new zone of SEP-enabled nodes by providing a directory and possibly relays or delegators, depending on the intended use case. In a nutshell, we improved the situation for the user and contributed an easy-to-implement option to the existing landscape for the developer or administrator.

The second design goal, Generality, emerged from the UNIX philosophy where every tool is intended to perform a single task well. This approach motivates creating several small tools, each implementing one task, in order to utilize their full strength when combined to perform a complex task. Sticking to this philosophy and its advantages for re-usability, we decided to create a modular toolset that focused on creating secure network connections.

As SEP is designed to be application agnostic, it enables legacy deployments to adapt its toolset and, most importantly, allows the emergence of new applications around the presented concepts. In order to demonstrate the strength of this modular approach, we implemented a small file sharing application that utilized all concepts presented in this paper, and a small information sharing application that only utilized the two building blocks of unique identities and node discovery. These first use cases easily reimplemented aspects of related work, such as *Magic Wormhole*, *Wirehub*, and *Syncthing*.

Practicability, the third design goal, aimed to increase the availability, interoperability, and robustness by utilizing the advantages of decentralization. We achieved this aim as generally all SEP enabled nodes could discover each other and, thus, attempt to initiate decentralized and secure peer-to-peer connections. Technically being centralized, however, directories can easily be added or modified without negative effects on other zones, which is a federated design comparable to similar techniques, such as Simple Mail Transfer Protocol (SMTP). Moreover, we relied on standard networking technologies, such as DNS, to further decentralize and reduce the load on single components, which further increased availability and robustness.

With the fourth design goal, Scalability, we targeted the administrator more than the user. By providing delegators, we developed a concept of trust management that allows administrators to easily maintain even a large number of nodes within multiple zones and possibly complex access rules in a secure manner. Moreover, adding additional relays, directories, or entire zones is straightforward and provides capacity that can be used by existing deployments. By supporting the concept of federation, we, thus, improved the situation for tools, such as *Syncthing* and *The Magic Wormhole*, where, by employing SEP, they can easily profit from redundant infrastructure components.

The fifth design goal, Security, is fundamentally based on the concept of identities and self-certified fingerprints. Once users have established trust by simply exchanging fingerprints, e.g., encoded in QR codes, mutually authenticated and encrypted end-to-end connections are established without further security-related decisions required to be taken by the user. Under the hood, this is realized by authenticated data distribution using signed record sets and the utilization of state-of-the-art security protocols with secure pre-defined settings. This not only applies to users of SEP-enabled applications, but also administrators or developers: SEP does not provide interfaces to change or disable the underlying cryptographic algorithms as with the libp2p project.

Table 3 summarizes the conclusion and contrasts the formulated design goals with the created toolset. From five design goals three are considered as reached. Two design goals still have room for improvement. For instance, the simplicity can be improved by modularizing SEP even more such that less infrastructure is required. Additionally, the generality of SEP can be further improved by providing a system level of integration such that established tools can benefit without modification. The reference [35] provides on overview of operating system level integration of TLS, which is comparable to such an effort.

## 9. Future Work

The presented concepts and the developed reference implementation potentially mark the beginning for new projects using this as a baseline. Especially interesting for future work is the topic of deferred messages, which deals with message delivering in case the target is offline. Considering the reusable and federated design of SEP, deferred messaging enables decentralized applications with built in end-to-end security where nodes might go online or offline at any time. Deferred messages might be implemented using techniques like the Double Ratchet Algorithm [36] in combination with public key distribution via the directory. Integrating such protocols at a lower level in SEP will surely enable novel application scenarios within a broad range of application domains, for instance data aggregation or distribution from mobile, embedded devices suffering from a weak internet link.

As implied, the integration of additional transports with different semantics will extend the applicability of the Stream Exchange Protocol. Due to its complexity, TLS has been suffering from security problems over the years, [31,37,38,39,40]. A modern and feature reduced transport block based on the Noise protocol framework [41], and more NAT traversal techniques will be a reasonable next step for the Stream Exchange Protocol.

In order to gain statistical performance data, it is planned to gather opt-in performance metrics from deployed SEP based applications within the context of different domains. Such data provides useful information of the used networking infrastructure and firewall configurations. Using such metrics, relevant performance parameters can be researched. To get a rough estimation of such analyses, the Syncthing project provides comparable data at [42].

Optimizing SEP for deployments in small and local networks without a directory instance is also of further interest. Nodes might distribute relevant information themselves using a secure local announcement technique extending protocols, such as Link Local Multicast Name Resolution (LLMNR) or Multicast DNS (mDNS), for local networks. Considering these glue points, we would be pleased to see future applications built on top of the presented building blocks, for instance secure P2P file sharing, media streaming applications, or embedded devices that expose their configuration endpoints via SEP. Last but not least, we encourage developers in creating bindings or implementations in various programming languages in order to make the concepts relevant for practical work.

## Figures and Tables

**Table 1:** Overview of the identified shortcomings.

	Complexity	Modularity	Maintainability	Scope	Insecurity
Syncthing		x	x	x	
Magic Wormhole		x	x	x	
libp2p	x				x
BitTorrent		x			x
WireHub		x			

**Table 2:** Overview of a directory record set. Entries marked with a an asterisk “*” are mandatory.

Data Type	Content
Address	protocol://ip-address:port
Relay	fingerprint
Blob	Placeholder for any binary data
Timestamp *	Time of creation
TTL *	Validity of record set in seconds
PubKey *	Public key of the announcing node
Signature *	Signature over the entire record set

**Table 3:** Overview of reached design goals. x: reached, (x): can be further improved.

Design Goal	Reached?	Comment
#1: Simplicity	(x)	Infrastructure components are required.
#2: Generality	(x)	Full system integration can be considered.
#3: Practicability	x	Concept leads to tools which are easy to use.
#4: Scalability	x	Scaling is realized via federation.
#5: Security	x	Strong default security settings are chosen.

## Data Availability

The source code implementing the presented concepts is available as Free Open Source (FOS) Software at [43].
